# Syphilis-attributable adverse pregnancy outcomes in China: a retrospective cohort analysis of 1187 pregnant women with different syphilis treatment

**DOI:** 10.1186/s12879-019-3896-4

**Published:** 2019-03-29

**Authors:** Huihui Liu, Niannian Chen, Jia Yu, Weiming Tang, Jianrong He, Huiyun Xiao, Suifang Lin, Fang Hu, Qiong Feng, Joseph D. Tucker, Huimin Xia, Xiu Qiu

**Affiliations:** 1 Division of Birth Cohort Study, Guangzhou Women and Children’s Medical Center, Guangzhou Medical University, 9 Jinsui Road, Guangzhou, 510623 China; 2 Department of Health Care, Guangzhou Women and Children’s Medical Center, Guangzhou Medical University, 9 Jinsui Road, Guangzhou, 510623 China; 3 Department of Obstetrics and Gynecology, Guangzhou Women and Children Medical Center, Guangzhou Medical University, 9 Jinsui Road, Guangzhou, 510623 China; 4 Institute for Global Health & Infectious Diseases, University of North Carolina at Chapel Hill, North Carolina, 27599 USA; 5 Faculty of Infectious and Tropical Diseases, London School of Hygiene and Tropical Medicine, Keppel Street, London, WC1E 7HT UK; 6 Department of Neonatal Surgery, Guangzhou Women and Children’s Medical Center, Guangzhou Medical University, 9 Jinsui Road, Guangzhou, 510623 China

**Keywords:** Syphilis, Treatment, Adverse outcomes, Pregnant women

## Abstract

**Background:**

Syphilis is responsible for a substantial burden of preventable adverse outcomes in pregnancy. The purpose of this study was to compare the frequency of adverse pregnancy outcomes among syphilis-seropositive women who received different treatment regimens at different times in Guangzhou, China.

**Methods:**

Pregnant women with syphilis infection who received prenatal and delivery services in Guangzhou between January 2014 and December 2016 were included. Association between treatment status and the composite adverse outcomes (preterm birth, infant smaller than gestational age, stillbirth, and spontaneous abortion) was estimated.

**Results:**

Of 1187 syphilis-seropositive pregnant women included in the analysis, 900 (75.8%) syphilis-seropositive pregnant women received treatment, and 287(24.2%) did not receive treatment. Adverse pregnancy outcomes were observed among 16.3% (147/900) of women with treatment and 33.8% (97/287) of women without treatment. Syphilis-seropositive pregnant women treated with one or two courses of penicillin had a similar risk of adverse pregnancy outcomes (adjusted RR = 1.36, 95% CI: 0.94–1.96). Adverse outcomes were more common among women whose non-treponemal serum test titer was >1:8 and received treatment after 28 weeks compared to before 28 weeks (adjusted RR = 2.34, 95% CI: 1.22–4.48).

**Conclusions:**

Women who received one course of penicillin and women who received two courses of penicillin had a similar risk of adverse pregnancy outcomes. Syphilis treatment before 28 weeks of pregnancy is critical. Strategies to promote high-quality prenatal services are needed.

## Background

Approximately one million pregnant women are infected with syphilis each year [1]. Many of these women do not receive testing and treatment, constituting a major missed public health opportunity. Syphilis in pregnancy causes stillbirth, neonatal death, prematurity, low birth weight, and congenital syphilis [2, 3].

As an epicenter of the global syphilis epidemic, China has a heavy burden of syphilis among pregnant women [4, 5]. Data from the Chinese national surveillance system and World Health Organization both suggest the prevalence of syphilis infection among pregnant women in China is between 0.3–1.0% [6–8]. For example, in 2013, the Chinese national surveillance system reported 15,884 cases of syphilis among pregnant women who delivered, 55.6% (8829) of these cases tested positive during pregnancy and 43.8% (6968) tested positive at labor [9]. To prevent mother-to-child transmission of syphilis, the Chinese government established the Integrated Prevention of Mother-to-Child Transmission (IPMTCT) system. The system provides opt-out, free syphilis, HIV, and hepatitis B testing at the first prenatal visit and at delivery [10, 11]. Women diagnosed with syphilis are provided free treatment. In addition, China has a comprehensive longitudinal maternal health system that accurately captures data describing adverse outcomes and treatment [12]. The city of Guangzhou has all medical institutions covered by the IPMTCT system and enhanced monitoring. This provides a strong opportunity for research to better understand syphilis treatment among pregnant women.

Previous research on maternal syphilis in China focused on the relatively rare outcome of congenital syphilis and not the large spectrum of adverse pregnancy outcomes known to be associated with syphilis infection [3]. Moreover, most of our knowledge about syphilis outcomes in pregnancy comes from small observational studies or from the pre-penicillin era [2, 13, 14]. The purpose of this study was to compare the frequency of adverse pregnancy outcomes among syphilis-seropositive women who received different treatment regimens at different times in Guangzhou, China.

## Methods

### Study design and participants

**Fig. 1:**
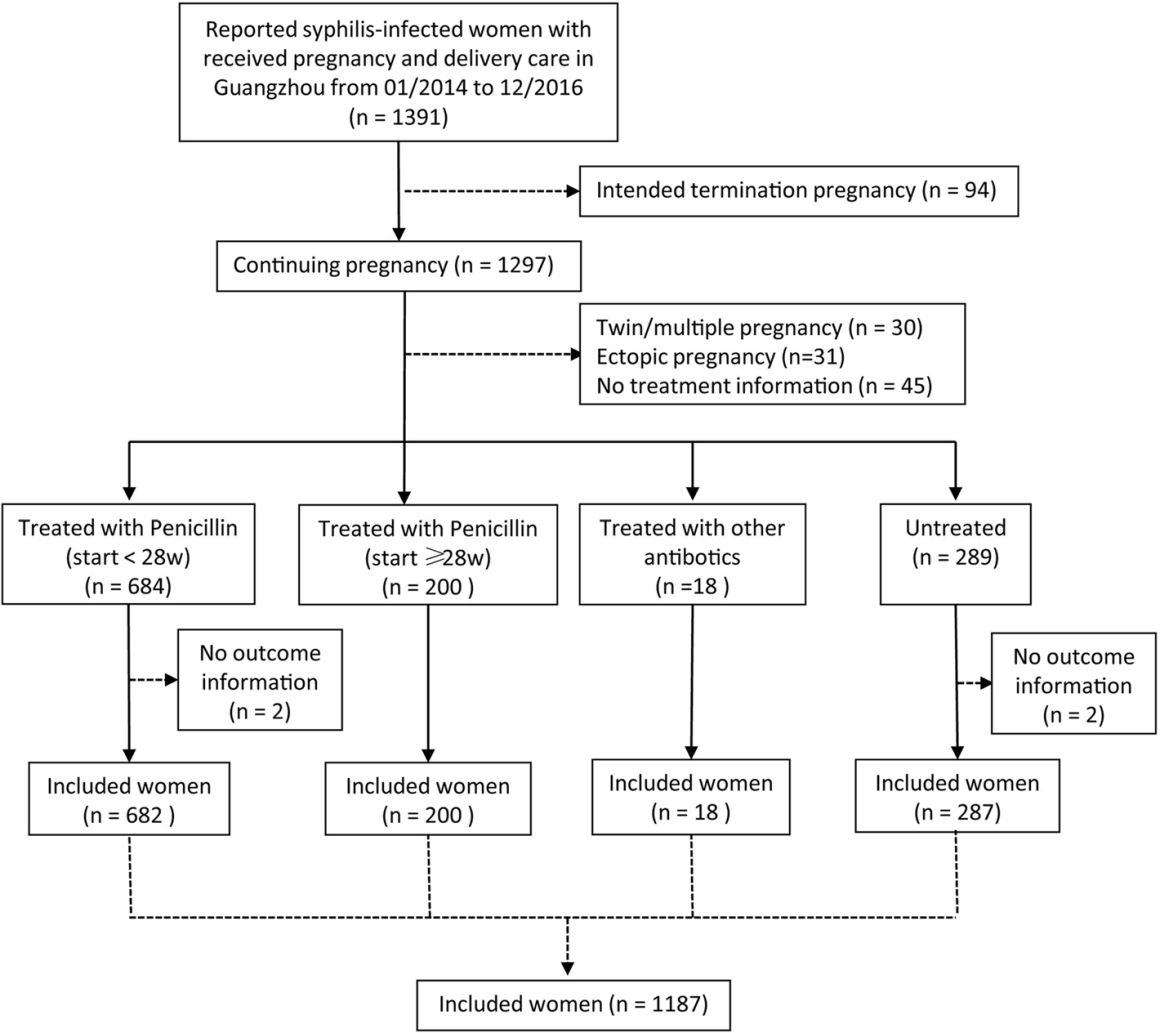
Sample size flow chart of study participants (Guangzhou, China, 2014–2016)

This retrospective cohort study used data within the Guangzhou IPMTCT system. Between January 2014 and December 2016, a total of 1391 (out of 7,009,069; 0.02%) pregnant women who received pregnancy and delivery care services in Guangzhou were diagnosed with syphilis and reported to the IPMTCT system. Syphilis-seropositive pregnant women who elected to terminate their pregnancy (*n* = 94), with ectopic pregnancy (*n* = 31), with twin or multiple gestation pregnancies (*n* = 30), with absent information to indicate if they received syphilis treatment or not (*n* = 45), and with incomplete outcome information (*n* = 4) were excluded. A total of 1187 women were included in this analysis (Fig. 1). The dataset of the Guangzhou IPMTCT system belong to the Guangzhou Women and Children’s Health Information Center. The data was anonymised and subsequently, consent requirement was waived and participatory consent was obtained by the Guangzhou Women and Children’s Medical Center Ethics Approval Board (2017072601).

### Diagnosis of syphilis in pregnancy

A diagnosis of syphilis required a positive treponemal test and a positive non-treponemal antibody test. Treponemal tests included treponema pallidum particle agglutination assay (TPPA) and enzyme linked immunosorbent assay (ELISA). Non-treponemal serum tests included toluidine red unheated serum test (TRUST) and rapid plasma reagin (RPR).

### Treatment for prevention of mother-to-child transmission of syphilis

According to national guidelines, syphilis-seropositive pregnant women should be provided with two courses of syphilis treatment for prevention of mother-to-child transmission [15]. A single complete course includes either benzathine penicillin 2.4 million units intramuscularly per week for three consecutive weeks or procaine penicillin 0.8 million units intramuscularly daily for 15 consecutive days. Ceftriaxone (1 g intravenous or intramuscularly daily for 10 consecutive days) may be used as an alternative to penicillin. For women who are allergic to penicillin, erythromycin (500 mg orally 4 times daily for 15 consecutive days) may be used.

The study divided participants into four groups based on syphilis treatment completed during pregnancy: (1) two courses: completed two full treatment courses during pregnancy, with an interval of more than one week between each other; (2) one course: completed at least one treatment course during pregnancy, but less than two full courses; (3) insufficient treatment: completed less than one full treatment course or patients who had information indicating they received treatment but without data confirming treatment completion; (4) no treatment. In addition, women were divided into four groups based on gestational age at treatment initiation and medication given: (1) penicillin treatment initiated before 28 weeks gestation; (2) penicillin treatment initiated at or after 28 weeks gestation; (3) non-penicillin treatment; (4) no treatment.

### Adverse outcomes and definitions

The primary outcome of the study was a composite adverse pregnancy outcome that included the following: (1) preterm birth: live birth delivered between 24 and < 37 weeks gestational age; (2) small for gestational age infant (SGA): birth weight < 10th percentile, based on the International Fetal and Newborn Consortium for the twenty-first Century criteria [16]; (3) stillbirth: fetal death at or after 28 weeks gestation or intrapartum death; and (4) spontaneous abortion: defined as spontaneous pregnancy loss before 28 weeks gestation. We chose not to include congenital infection in this manuscript for the following reasons: many clinics lack the knowledge and diagnostics required to make an accurate congenital syphilis diagnosis, resulting in misclassification bias [17]; stillbirth, preterm birth, and the other adverse outcomes are responsible for substantial morbidity according to disability-adjusted life-year estimates [18, 19]; other studies have focused on congenital syphilis and maternal interventions in China [3].

Information on pregnancy outcomes was reported by the hospital where participants gave birth. Delivery data (including delivery date, gestational age, gender, birth weight, live birth status) were obtained through the electronic Guangzhou Perinatal Health Care and Delivery Surveillance System (GPHCDSS). The Guangzhou database was started in 2000 and covers 99% all delivering mothers and infants in Guangzhou. Information from the IPMTCT and GPHCDSS databases can be linked through matching maternal ID and name [16]. In both systems, gestational age at birth was expressed as completed weeks and was based on first- or second-trimester ultrasound. In the absence of a recorded ultrasound data, last menstrual period was used to calculate gestational age.

### Statistical analysis

Descriptive analysis was used to summarize socio-demographic information of study participants. Chi-squared tests were used to compare socio-demographic information and clinical characteristics of participants. T-test was only used to analyze age. Non-penicillin treatment cases were described, but not further analyzed because of small sample sizes. Binomial regression models were used to analyze correlations of syphilis-seropositive pregnant women receiving different treatment regimens and adverse pregnancy outcomes, and with different non-treponemal serum test titers and adverse pregnancy outcomes, with corresponding relative risk (RR) and 95% confidence intervals (CI) reported and adjusted for potential confounders (maternal age, marital status, household registration, parity, treatment status and treatment start time during pregnancy). We further examined the relationships between different treatments and adverse pregnancy outcomes, stratifying for non-treponemal serum test titer. All analyses were conducted using SAS version 9.3 (SAS Institute, Cary, USA). Significance was set as *α* = 0.05.

## Results

### Participants

Among the included 1187 participants, 244 (21.2%) of participants had at least one composite adverse pregnancy outcome, including 17 (1.4%) spontaneous abortions, 24 (2.0%) still births, 125 (10.5%) preterm births, and 86 (7.2%) SGAs (Table 1).

**Table 1:** Adverse pregnancy outcomes of study participants in Guangzhou, China, 2014–2016

Adverse pregnancy outcomes	N (%)
No	943 (78.8)
Yes	244 (21.2)
Spontaneous abortion	17 (1.4)
Still birth	24 (2)
Preterm birth	125 (10.5)
SGA^a,b^	86 (7.2)

Abbreviations: *SGA* small for gestational age infant

^a^Based on INTERGROWTH-21st criteria

^b^Including 8 preterm births

**Fig. 2:**
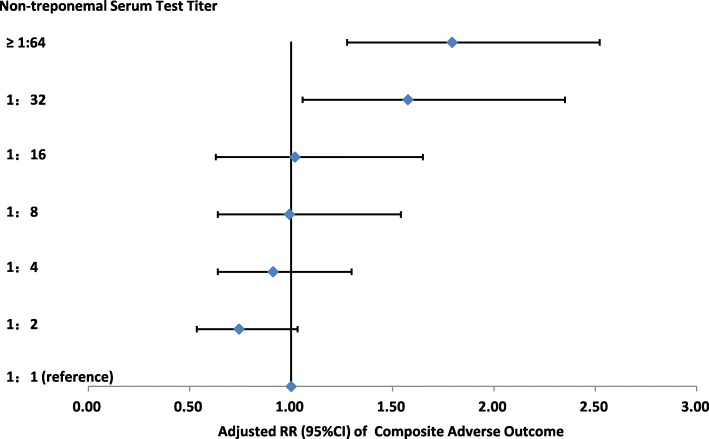
Risk of composite adverse outcome among syphilis-seropositive pregnant women by non-treponemal serum test titers (Guangzhou, China, 2014–2016)

Figure 2 shows the dose-response of non-treponemal serum test titers and adverse outcomes among syphilis-seropositive pregnant women in Guangzhou. Maternal age, marital status, household registration, parity and treatment status were adjusted in this model. Given the size of subgroups, we grouped the non-treponemal serum test titers variable into “≤ 1:8” and “>1:8”. Women whose non-treponemal serum test titers were >1:8 had higher rate of adverse outcome compared to women with titers ≤1:8 (adjusted RR = 1.60, 95% CI: 1.25–2.05).

Participant characteristics were compared between women with and without adverse outcomes (Table 2). The mean age of these participants was 30.2 (standard deviation 5.9) years old. The majority of the participants were migrants (839, 70.6%) and diagnosed in a public hospital (1016, 85.6%). 402 (33.9%) participants were diagnosed with syphilis at or after 28 weeks gestation, of which 200 (16.8%) were diagnosed at delivery. Among the included 1187 women, 682 (57.4%) initiated penicillin treatment before 28 weeks gestation, 200 (16.8%) initiated treatment at or after 28 weeks gestation, 18 (1.5%) received non-penicillin treatment, and 287 (24.2%) did not receive any treatment during pregnancy. The median gestational age at delivery for live births was 39 weeks. The age of syphilis-seropositive pregnant women with adverse pregnancy outcomes was lower than those without adverse pregnancy outcomes (29.1 vs 30.5, *p* < 0.001). Syphilis-seropositive pregnant women who were unmarried, diagnosed at or after 28 weeks of pregnancy, going to the suburban hospital and with no treatment were more likely to have adverse outcomes (*p* < 0.05).

**Table 2:** Participant characteristics compared across syphilis-seropositive pregnant women with and without adverse pregnancy outcomes in Guangzhou, China, 2014–2016

Variables	All women N	Women without adverse outcomes N (%)	Women with adverse outcomes N (%)	χ^2^	*p*
Age (Mean, SD, years)	30.2 ± 5.9	30.5 (5.8)	29.1 (6.4)	3.340^a^	< 0.001
Household registration					
Local	348	284 (81.6)	64 (18.4)	1.413	0.235
Migrant	839	659 (78.6)	180 (21.5)		
Marital status					
First marriage	944	775 (82.1)	169 (17.9)	26.466	< 0.001
Unmarried	136	86 (63.2)	50 (36.8)		
Others	107	82 (76.6)	25 (23.4)		
Public hospital					
Yes	1016	831 (81.8)	185 (18.2)	0.055	0.815
No	171	112 (65.5)	59 (34.5)		
Hospital area					
Urban	383	308 (80.4)	75 (19.6)	8.481	0.014
Suburban	417	313 (75.1)	104 (24.9)		
Rural	387	322 (83.2)	65 (16.8)		
Time of syphilis diagnosis					
< 28 weeks of gestation	785	655 (83.4)	130 (16.6)	22.659	< 0.001
≥ 28 weeks of gestation	402	288 (71.6)	114 (28.4)		
Syphilis infection stage					
Latent	958	755 (78.8)	203 (21.2)	1.375	0.503
Stage I-II	72	58 (80.6)	14 (19.4)		
Unknown	157	130 (82.8)	27 (17.2)		
Non-treponemal serum test titer					
≤ 1:8	1016	831 (81.8)	185 (18.2)	23.796	< 0.001
>1:8	171	112 (65.5)	59 (34.5)		
Treatment status					
No treatment	287	190 (66.2)	97 (33.8)	40.863	< 0.001
Penicillin treatment < 28 weeks gestation	682	573 (84)	109 (16)		
Penicillin treatment ≥28 weeks gestation	200	165 (82.5)	35 (17.5)		
Non-penicillin treatment	18	15 (83.3)	3 (16.7)		

Abbreviations: *SD* standard deviation

^a^T-test

### Relative risk of adverse pregnancy outcomes by treatment status

Associations between syphilis treatment regimens and the composite adverse pregnancy outcomes are shown in Table 3. Two-hundred forty-one women had an adverse pregnancy outcome. Among these women, 109 (16.0%) were treated with penicillin before 28 weeks gestation, 35 (17.5%) were treated with penicillin at or after 28 weeks gestation, and 97 (33.8%) were untreated. After adjusting for covariates, adverse outcomes were similar among the women treated with penicillin before 28 weeks gestation and those treated at or after 28 weeks gestation (adjusted RR = 1.13, 95% CI: 0.79–1.61, *p* = 0.471). Adverse outcomes were significantly more common among those without treatment compared to those treated with penicillin before 28 weeks gestation (adjusted RR = 2.07, 95% CI: 1.59–2.68). After adjusting for covariates, when compared to those treated with two courses of penicillin, adverse outcomes were similar among the women treated with one course of penicillin (adjusted RR = 1.36, 95% CI: 0.94–1.96).

**Table 3:** Risk of composite adverse outcomes among syphilis-seropositive pregnant women by syphilis treatment status in Guangzhou, China, 2014–2016^a^

Treatment during pregnancy	Women with adverse outcomes, N (%)	Crude RR (95% CI)	Adjusted RR (95% CI)
Treatment initiation			
Penicillin <28w	109 (16)	reference	reference
Penicillin ≥28w	35 (17.5)	1.10 (0.77–1.55)	1.13 (0.79–1.61) ^b^
No treatment	97 (33.8)	2.11 (1.67–2.68)	2.07 (1.59–2.68) ^b^
Treatment course			
2 courses	73 (14.2)	reference	reference
1 course	38 (17.8)	1.26 (0.88–1.80)	1.36 (0.94–1.96)^c^
Insufficient treatment	33 (21.3)	1.50 (1.04–2.17)	1.58 (1.08–2.31)^c^
No treatment	97 (33.8)	2.38 (1.82–3.11)	–

Abbreviations: *RR* relative risk, *CI* confidence intervals

^a^None-penicillin treatment cases were excluded

^b^Adjusted for age, marital status, household registration, and multipara status

^c^ Adjusted for age, marital status, household registration, multipara status, and the start time of treatment in the gestation

Among participants with non-treponemal serum test titers ≤1:8, after adjusting for covariates, adverse outcome was similar between women treated at or after 28 weeks gestation compared to those treated before 28 weeks gestation (adjusted RR = 0.91, 95% CI: 0.60–1.38) (Table 4). Among participants with non-treponemal serum test titer >1:8, after adjusting for covariates, incidence of adverse outcomes was significantly higher among those treated at or after 28 weeks gestation compared to those treated with penicillin before 28 weeks gestation (adjusted RR = 2.34, 95% CI: 1.22–4.48).

**Table 4:** Risk of composite adverse outcome among syphilis-seropositive pregnant women with different non-treponemal serum test titers by syphilis treatment status in Guangzhou, China, 2014–2016 ^a^

Treatment during pregnancy	Women with adverse outcomes, N (%)	Crude RR (95% CI)	Adjusted RR (95% CI)
Non-treponemal serum test titers ≤1:8 (*N* = 999)			
Treatment initiation			
Penicillin < 28w	95 (10.6)	reference	reference
Penicillin ≥28w	25 (34.6)	0.88 (0.59–1.33)	0.91 (0.60–1.38)^b^
No treatment	63 (41.7)	1.73 (1.31–2.28)	1.77 (1.31–2.40)^b^
Treatment course			
2 courses	67 (14.9)	reference	reference
1 course	31 (16.2)	1.09 (0.74–1.61)	1.21 (0.82–1.81)^c^
Insufficient treatment	22 (16.9)	1.14 (0.73–1.77)	1.22 (0.78–1.91)^c^
No treatment	63 (27.6)	1.86 (1.37–2.52)	–
Non-treponemal serum test titers >1:8 (*N* = 170)			
Treatment initiation			
Penicillin <28w	14 (15.9)	reference	reference
Penicillin ≥28w	10 (43.5)	2.73 (1.40–5.34)	2.34 (1.22–4.48)^b^
No treatment	34 (57.6)	3.62 (2.14–6.14)	2.93 (1.66–5.17)^b^
Treatment course			
2 courses	6 (14.7)	reference	reference
1 course	7 (31.3)	3.39 (1.28–9.02)	2.19 (0.90–5.31)^c^
Insufficient treatment	11 (50.0)	4.69 (1.95–11.32)	2.40 (1.05–5.52)^c^
No treatment	34 (63.4)	6.15 (2.78–13.58)	–

Abbreviations: *RR* relative risk, *CI* confidence intervals

^a^None-penicillin treatment cases were excluded

^b^Adjusted for age, marital status, household registration, and multipara status

^c^Adjusted for age, marital status, household registration, multipara status, and the start time of treatment in the gestation

## Discussion

This retrospective cohort study examined associations between syphilis treatment regimens and adverse pregnancy outcomes among pregnant women with syphilis. We found that adverse outcomes were more common among women with non-treponemal serum test titers >1:8 who received penicillin treatment after 28 weeks of pregnancy. We observed syphilis-seropositive pregnant women who received one or two courses of penicillin treatment had similar rates of adverse pregnancy outcomes. Our study advances the literature by examining a composite adverse outcome (instead of a single outcome) with a large sample size, integrating two high-quality data sources, and leveraging China’s extensive maternal child health system.

Our study has shown that adverse outcomes were more common among those whose non-treponemal serum test titer was >1:8 and received penicillin treatment after 28 weeks of pregnancy. This suggests that one course of penicillin before 28 weeks of pregnancy is critical for preventing adverse outcomes of syphilis, while there is the potential for *T. pallidum* transmission from mother to child as early as 9–10 weeks gestation [20], our data suggest that treatment before 28 weeks gestation can avert a large burden of adverse outcomes.

Compared to two courses of treatment, one course of penicillin had similar rates of adverse outcomes in this study. Chinese national guidelines recommend two courses of intramuscular benzathine penicillin, with each course consisting of one shot per week for three weeks [15]. However, national guidelines are often not followed in clinical practice. Our study found only half of pregnant women treated for syphilis received treatment that met national guidelines. Although our study was not designed to establish non-inferiority between one and two courses, it suggests that one course may be reasonable, at least for preventing adverse outcomes included in our study.

Our data also suggest most syphilis-seropositive pregnant women were migrant women. Migrant women have worse pregnancy outcomes compared to pregnant women from Guangzhou. Our study is consistent with previous literature showing worse pregnancy outcomes among migrants in China and Italy [21–23]. The lack of or inability to access services may explain why migrant pregnant women with syphilis in our study had delayed screening and lower treatment rates. Poverty, lack of health insurance, and inability to recoup health insurance benefits in large cities may be barriers to receiving ANC services for migrant pregnant women [23, 24]. Strategies to improve access to pregnancy care, guarantee similar quality of ANC services across different settings, expand insurance coverage, and promote syphilis testing may be useful to address this problem.

There are several limitations in this study. First, we only included data from a single large city. Guangzhou is the provincial hub and has greater resources and better infrastructure for screening and treating syphilis in pregnancy than most other settings in China. Second, although all women were screened for HIV infection, we do not have data describing other sexually transmitted co-infections which could also cause adverse outcomes in pregnancy. Third, pregnant women in China often receive care at several hospitals and by multiple physicians. As a result, making inferences about the influence of specific hospitals or physicians is challenging. However, in this study, we successfully linked all reported cases. Fourth, few women were treated with ceftriaxone, a drug that may be useful for women who are allergic to penicillin [25]. Fifth, initial infection and re-infection cannot be differentiated based on syphilis serology alone, and therefore our study could not make inferences regarding re-infection rates among pregnant women. Finally, we did not differentiate outcomes based on when patients were diagnosed.

This study has several research and policy implications. Further implementation research is needed to improve screening and expand treatment. Quality improvement measures to enhance early syphilis testing and timeliness of treatment following early syphilis testing among all women should be further investigated. Given the inherent ethical dilemmas of studies with comparator arms, further cohort research may be most appropriate. Modeling research to suggest particular policy strategies may also be useful. At the policy level, packages of services for women, especially for migrant women, are needed. Strategies to simplify and better integrate current syphilis treatment programs are also required.

## Conclusions

Our study reveals adverse outcomes were similar between syphilis-seropostive pregnant women who received one or two courses of penicillin treatment and treatment before 28 weeks gestation can avert a large burden of adverse outcomes. The results of our study have practical implications for the prevention and treatment syphilis-attributable adverse pregnancy outcomes. Strategies are warranted to promote higher-quality, more comprehensive prenatal healthcare services.

## Abbreviations

ANC: Antenatal Care
CI: Confidence intervals
GPHCDSS: Guangzhou Perinatal Health Care and Delivery Surveillance System
IPMTCT: Integrated Prevention of Mother-to-Child Transmission
RR: Relative risk
SGA: Small for gestational age infant
